# Changes in agricultural carbon emissions and factors that influence agricultural carbon emissions based on different stages in Xinjiang, China

**DOI:** 10.1038/srep36912

**Published:** 2016-11-10

**Authors:** Chuanhe Xiong, Degang Yang, Fuqiang Xia, Jinwei Huo

**Affiliations:** 1 Xinjiang Institute of Ecology and Geography, Chinese Academy of Sciences, Urumqi Xinjiang 830011, China; 2 University of Chinese Academy of Sciences, Beijing 100049, China

## Abstract

Xinjiang’s agricultural carbon emissions showed three stages of change, i.e., continued to rise, declined and continued to rise, during 1991–2014. The agriculture belonged to the “low emissions and high efficiency” agriculture category, with a lower agricultural carbon emission intensity. By using the logarithmic mean divisia index decomposition method, agricultural carbon emissions were decomposed into an efficiency factor, a structure factor, an economy factor, and a labour factor. We divided the study period into five stages based on the changes in efficiency factor and economy factor. Xinjiang showed different agricultural carbon emission characteristics at different stages. The degree of impact on agricultural carbon emissions at these stages depended on the combined effect of planting-animal husbandry carbon intensity and agricultural labour productivity. The economy factor was the critical factor to promote the increase in agricultural carbon emissions, while the main inhibiting factor for agricultural carbon emissions was the efficiency factor. The labour factor became more and more obvious in increasing agricultural carbon emissions. Finally, we discuss policy recommendations in terms of the main factors, including the development of agricultural science and technology (S&T), the establishment of three major mechanisms and transfer of rural labour in ethnic areas.

Global warming caused by carbon emissions has resulted in significant adverse effects on human economic and social development^1^. It has become the common goal of countries worldwide to address climate change, reduce carbon emissions, and implement sustainable development strategies. China has proposed that by 2030, its CO_2_ emissions per unit of GDP will have decreased by 60–65% compared with emissions in 2005, and this has been a binding target incorporated into the national economic, social development and long-term planning.

In addition to the second and third industries, agriculture is also an important source of carbon emissions^2^. Carbon emissions from the agricultural ecosystem account for 17% of China’s total greenhouse gas emissions, which is approximately 4% higher than the proportion of the world^3^,^4^.

Xinjiang is located in the northwest of China, secluded in the latitude of Eurasia hinterland, with a total area of 1.66 × 10^6^ km^2^. Xinjiang is the largest province in the area of China and borders eight countries: Russia, Kazakhstan, Kyrgyzstan, Tajikistan, Pakistan, Mongolia, India and Afghanistan. Xinjiang is China’s largest production base of grain, cotton, fruit and livestock, which meets China’s growing consumer demand for agricultural products. This also produces a huge agricultural carbon emission and puts great pressure on the environment. Meanwhile, Xinjiang is located in the typical drylands of China, and the carbon emissions from the drylands soil have a close relationship with global warming^5^. Moreover, together with the fragile natural environment, ecological damage is irreversible^6^. Therefore, studying changes about agricultural carbon emissions and the factors that influence agricultural carbon emissions at different stages, as well as exploring the driving factors for agricultural carbon emissions in Xinjiang can provide important theoretical insights for environment conservation, technology innovation and the low-carbon agricultural economy development.

The logarithmic mean divisia index (LMDI) is a very effective method to study the factors that influence carbon emissions^7^,^8^,^9^. Only a few studies have applied the decomposition method to analyse agricultural carbon emissions^2^,^10^.

From the perspective of different stages, some researchers paid close attention to agricultural carbon emissions and divided the study period into sub-periods based on the trend of agricultural carbon emissions and the decoupling relationship between agricultural carbon emissions and agricultural economic growth^11^,^12^,^13^,^14^.

Through the above overview of the literature, most previous studies divided stages mainly based on the trend of total agricultural carbon emissions and the decoupling relationship between agricultural carbon emissions with the agricultural economic growth without considering different agricultural carbon emission characteristics. Because Xinjiang shows different carbon emission characteristics in different stages of agricultural economic development, it is necessary to divide the agricultural economy development stage according to the different agricultural carbon emission characteristics and analyse the development trend and influencing factors of each stage using time series data. In this paper, we explore the effects of various stages on the promotion and suppression of agricultural carbon emissions by decomposition of agricultural carbon emissions at different stages. Therefore, the analysis results can provide effective agricultural carbon reduction proposals for policy makers.

## Material and Methods

### Agricultural carbon emission calculation

Agricultural greenhouse gas emissions mainly come from the use of agricultural land, paddle field and livestock farming^2^,^10^,^15^,^16^,^17^.

In accordance with the carbon emission equation of some researchers^2^,^10^,^18^, we construct the agricultural carbon emissions formula as follows:


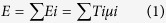


where *E* is the total agricultural carbon emissions, *E*_*i*_ is the agricultural carbon emissions of agricultural carbon source *i*, *T*_*i*_ is the amount of agricultural carbon source *i* and *μ*_*i*_ is the agricultural carbon emission coefficient of agricultural carbon source *i*. Three types of carbon emissions (C, CH_4_ and N_2_O) have been calculated in this paper and we convert CH_4_ and N_2_O to standard carbon according to IPCC^19^. All of the carbon sources are from the research of Jane M F Johnson, Tian Yun and Xiong Chuanhe^2^,^10^,^15^. All coefficients in this paper are from the research of Xiong Chuanhe^2^.

### Decomposition of agricultural carbon emissions impact factors

This research will apply the LMDI decomposition method to decompose Hotan’s agricultural carbon emissions. The LMDI method can make the model more convincing, which has the advantage of being able to be used for the decomposition of incomplete data sets and benefits from the lack of an unexplainable residual in the results. This can be used for either multiplicative or additive decompositions, where multiplicative decompositions decompose the ratio change of the factors and additive decompositions decompose the amount of change in the factors^2^,^7^,^8^,^9^,^10^,^20^,^21^,^22^,^23^,^24^,^25^,^26^.

In accordance with the existing literature in past research^2^,^7^,^8^,^9^,^10^,^20^,^21^,^22^,^23^,^24^,^25^,^26^ and combined with the actual situation of the agricultural carbon emissions, we establish the formula:


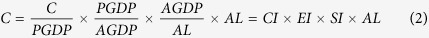


where *C*, *PGDP*, *AGDP*, and *AL* represent agricultural carbon emissions, planting-animal husbandry gross output value, agricultural gross output value, and employment labour of the agricultural industry, respectively. *CI, EI, SI* and *AL* represent efficiency factor, structure factor, economy factor and labour factor respectively.

- The efficiency factor (denoted by *ΔCI*) reflects the changes in agricultural carbon emissions per unit of planting-animal husbandry gross output value;
- The structure factor (denoted by *ΔEI*) reflects the changes in the relative share of the planting-animal husbandry gross output value in total agricultural gross output value;
- The economy factor (denoted by *ΔSI*) reflects the changes in agricultural economic output per unit labour input;
- The labour factor (denoted by *ΔAL*) reflects changes in the labour input.

Since various types of carbon source are closely related to the planting industry and animal husbandry, the agricultural carbon emissions we studied in this paper is the actual carbon emissions from farming and livestock production. Since there are great inconsistencies in the departments of agriculture production and scale quantitative method, we make uniform output as a comparative amount in order to make the comparison convenient.

In Equation (2), the total agricultural carbon emissions of the base period and the T period were set as *C*_*0*_ and *C*_*t*_, and *ΔC*_*tot*_ is the change in agricultural carbon emissions from the base period to period t. By applying additive decomposition, the agricultural carbon emission factors can be decomposed as follows^2^:


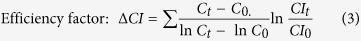



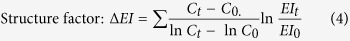



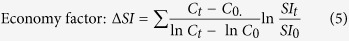



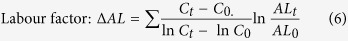


The total effect is: 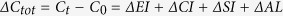.

### Data description

The data in this study come from the Xinjiang Statistical Yearbook (1992–2015) and the China Rural Statistical Yearbook (1992–2015). Taking into account that the agricultural production values, which were calculated with respect to actual price, cannot be compared longitudinally, we use the added value of agricultural comparable price (like comparable price of GDP), i.e., the price in 1990, as the price of the benchmark year.

## Results

### Changes in agricultural carbon emission

Formula (1) was applied to calculate the agricultural carbon emissions in Xinjiang from 1991 to 2014 and to calculate parameters such as the amount of agricultural carbon emissions per 10000 CNY agricultural output value (agricultural carbon emission intensity).

### Change in the total amount of agricultural carbon emissions

**Figure 1:**
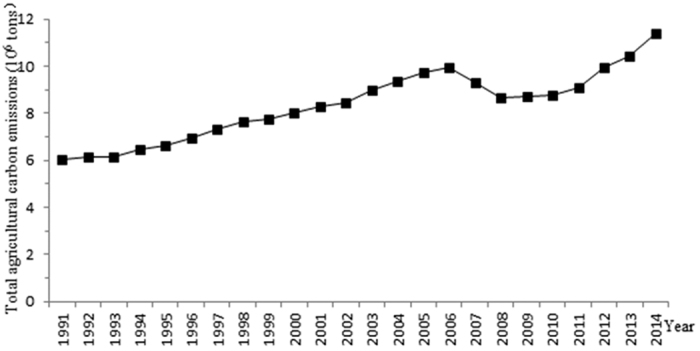
Changes in total agricultural carbon emissions in Xinjiang. The x-axis is the central year of the 24-year moving window from 1991–2014. The y-axis is the total agricultural carbon emissions whose unit is 10^6^ tons.

From 1991–2014, Xinjiang’s agricultural carbon emissions showed three stages of change, i.e., continued to rise, declined and continued to rise, during this period (Fig. 1). In the first stage, from 1991 to 2006, carbon emissions continued to rise. In the second stage, from 2007 to 2010, carbon emissions decreased. In the third stage, from 2011 to 2014, carbon emissions continued to rise. Xinjiang’s agricultural carbon emissions exhibited an overall increasing trend from 6.04 × 10^6^ tons to 11.42 × 10^6^ tons, which represented an 89.07% increase over the 24 years.

### Structural changes in agricultural carbon emissions

Xinjiang is the base of planting and animal husbandry in China. Agricultural carbon emissions from planting and animal husbandry accounted for more than 99.8% of the total agricultural carbon emissions during the study years, whereas carbon emissions produced during the growth and development of rice were less than 0.20%, which can be ignored in this part.

**Figure 2:**
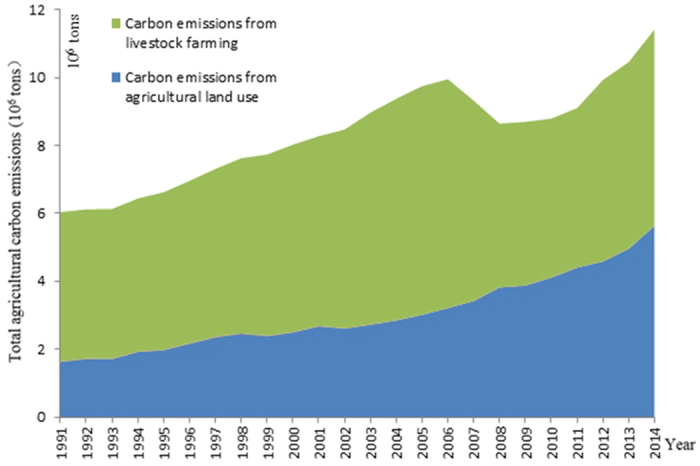
Changes in agricultural carbon emissions from agricultural land use and livestock farming in Xinjiang from 1991–2014. Changes in carbon emissions from agricultural land use are marked in blue, and changes in carbon emissions from livestock farming are marked in yellow.

**Figure 3:**
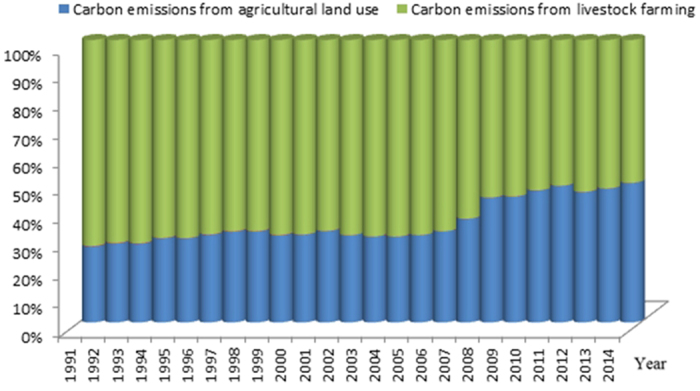
Proportion changes in agricultural carbon emissions from agricultural land use and livestock farming in Xinjiang from 1991–2014. Proportion changes in carbon emissions from agricultural land use are marked in blue, and proportion changes in carbon emissions from livestock farming are marked in yellow.

In 1991, Xinjiang’s agricultural carbon emissions were based on planting and animal husbandry in proportions of 26.86% (162.21 × 10^4^ tons) and 72.93% (440.40 × 10^4^ tons), respectively (Figs 2 and 3). The proportion of animal husbandry gradually declined thereafter until 2008, when the proportions of planting and animal husbandry were 49.33% (563.38 × 10^4^ tons) and 50.60% (577.96 × 10^4^ tons), respectively (Figs 2 and 3). This structure has been continuously optimized.

### Agricultural carbon emission intensity

**Figure 4:**
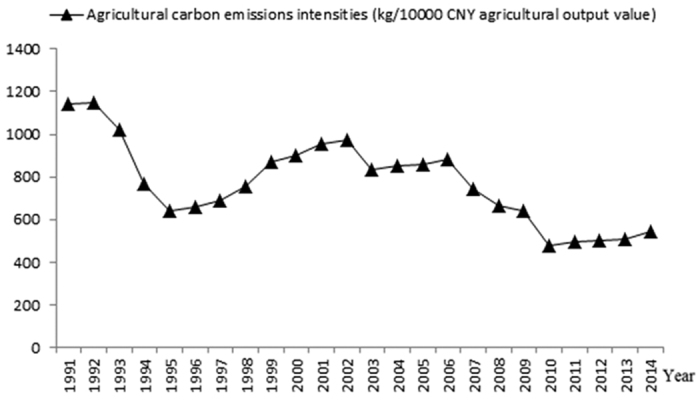
Changes in Xinjiang’s agricultural carbon emissions intensity during 1991–2014. Agricultural carbon emissions intensity = the total agricultural carbon emissions/agricultural output value; its unit is kg/10^4^ CNY.

From 1991–2014, Xinjiang’s agricultural carbon emission intensity showed three stages of change, i.e., decline, rise and decline. In the first stage, from 1991 to 1995, carbon emissions declined. In the second stage, from 1996 to 2002, carbon emissions increased. In the third stage, from 2003 to 2014, carbon emissions decreased in a fluctuating manner (Fig. 4). With the development of science and technology, the efficiency improved continuously, so the agricultural carbon emission intensity was reduced.

### Factors that influence agricultural carbon emissions based on different stages

**Figure 5:**
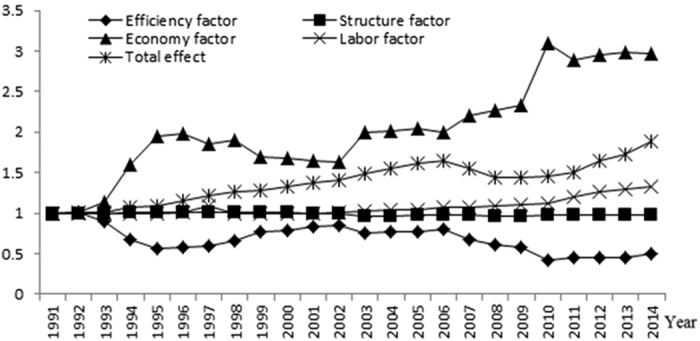
Decomposition of agricultural carbon emissions in Xinjiang during 1991–2014. The x-axis is the central year of the 24-year moving window from 1991–2014, and the y-axis is the ratio change.

Through the multiplicative LMDI decomposition, we obtain the results shown in Fig. 5. Among these factors, the economy factor and the efficiency factor changed greatly. The economy factor fluctuated between 1.015 and 3.095, which generally promoted agricultural carbon emissions, whereas the efficiency factor fluctuated between 0.426 and 1.005, which generally inhibited agricultural carbon emissions. The structure factor and the labour factor changed a little, which had less impact on agricultural carbon emissions. Through Fig. 5 we can see that the main factor driving agricultural carbon emissions was the economy factor, whereas the main factor inhibiting carbon emissions was the efficiency factor. The economic factor and the efficiency factor determined the development trend of the total effect. Therefore, we can divide the study period (1991–2014) into five stages that are mainly based on the changes in Xinjiang’s agricultural economic output per unit labour input and planting-animal husbandry carbon intensity: 1991–1995, high efficiency and high output; 1996–2002, low efficiency and low output; 2003–2006, low efficiency and high output; 2007–2010, high efficiency and high output; 2011–2014, low efficiency and high output.

### High efficiency and high output (1991–1995)

At this stage, the efficiency factor curbed the agricultural greenhouse gas emissions, and the inhibitory effect was enhanced. This showed that the effect of the efficiency factor was higher during this stage, and the promoting effect of the economic factor on agricultural carbon emissions was increasing, indicating that the effect of the economy factor was higher during this stage. Therefore, the main features of this stage are high efficiency and high output. The main reason is that this stage is in the period of the development of agriculture after reform and opening up, so Xinjiang’s agricultural economy and agricultural science and technology developed rapidly.

Regarding the factors that influence agricultural carbon emissions, the efficiency factor was the main inhibitory factor for agricultural carbon emissions (Table 1), which achieved 68.72% (369.91 × 10^4^ tons) agricultural carbon emissions reduction over the study period (1991–2014). The labour factor decreased the agricultural carbon emissions by 1.17 × 10^4^ tons (Table 1), which cut 0.22% of the total agricultural carbon emissions increment over the study period (1991–2014).

The economy factor is the most contributing factor to the increase of agricultural carbon emissions, which increased by 78.86% (424.46 × 10^4^ tons) of the total agricultural carbon emissions increment over the study period (1991–2014). The structure factor increased carbon emissions by 5.15 × 10^4^ tons, and the contribution of this effect to the total agricultural carbon emissions increment over the study period (1991–2014) was 0.96% (Table 1).

### Low efficiency and low output (1996–2002)

At this stage, the efficiency factor promoted agricultural carbon emissions, and this indicates that the effect of the efficiency factor was rather low at this stage. The economy factor had a decreasing effect on promoting agricultural carbon emissions, even though it curbed agricultural carbon emissions in 1997, 1999 and 2001 indicating that agricultural economic growth decreased during this stage. Therefore, the main features of this stage were low efficiency and low output. The main reason is that agricultural products were difficult to sell and Xinjiang’s agricultural economy had been a recession, which reduced the enthusiasm of farmers.

The performance of each factor at this stage is opposite to that of the first stage. The economy factor is the main inhibitory factor for agricultural carbon emissions (Table 2), which achieved 13.14% (70.72 × 10^4^ tons) agricultural carbon emissions reduction of the total agricultural carbon emissions increment over the study period (1991–2014). The structure factor decreased the agricultural carbon emissions by 6.84 × 10^4^ tons (Table 2), which cut 1.27% of the total agricultural carbon emissions increment over the study period (1991–2014). The efficiency factor is the most contributing factor to the increase of agricultural carbon emissions, which increased by 47.50% (255.70 × 10^4^ tons) of the total agricultural carbon emissions increment over the study period (1991–2014). The labour factor increased carbon emissions by 6.62 × 10^4^ tons, and the contribution of this effect to the total carbon emissions increment over the study period (1991–2014) was 1.23% (Table 2).

### Low efficiency and high output (2003–2005)

At this stage, the efficiency factor had a decreasing effect on curbing agricultural carbon emissions, even though it promoted agricultural carbon emissions in 2004 which indicates that the effect of the efficiency factor was relatively lower during this stage. Meanwhile, the promoting effect of the economic factor on agricultural carbon emissions was increasing, meaning that the effect of the economy factor was higher during this stage. Therefore, the main features of this stage are low efficiency and high output. The reason is that China carried out the policy of the development in western China and the development policy of agriculture, countryside and farmers in this stage, Xinjiang’s agricultural economy developed rapidly, but the development of agricultural science and technology was slow.

The efficiency factor only decreased agricultural carbon emissions by 87.45 × 10^4^ tons, far below that in the stage of high efficiency and high growth (1991–1995). The structure factor decreased carbon emissions by 17.77 × 10^4^ tons (Table 3), which cut 3.30% of the total agricultural carbon emissions increment over the study period (1991–2014), meaning the agricultural production structure had improved in this stage. The economy factor increased by 37.39% (201.25 × 10^4^ tons) of the total agricultural carbon emissions increment over the study period (1991–2014), indicating the agricultural economy had been restored. The labour factor increased carbon emissions by 31.73 × 10^4^ tons, and the contribution of this effect to the total carbon emissions increment over the study period (1991–2014) was 5.89% (Table 3), indicating that the labour factor was an important factor to promote agricultural carbon emission in this stage.

### High efficiency and high output (2006–2010)

At this stage, the efficiency factor had an increasing effect on curbing agricultural carbon emissions and this indicated that the effect of the efficiency factor was higher at this stage, and the economy factor had an increasing effect on promoting agricultural carbon emissions, indicating that agricultural economic growth increased during this stage. Therefore, the main features of this stage are high efficiency and high output. We describe the main reasons for this effect. On the one hand, China abolished the agricultural tax in 2006, which increased the enthusiasm of farmers. On the other hand, due to the diversified development of grain, cotton, fruit and livestock, Xinjiang’s agricultural production structure had been further optimized and Xinjiang’s agricultural economic growth entered a new round of rapid growth period.

This stage is similar to the first stage (1991–1995). Table 4 shows that the efficiency factor achieved a 78.82% (424.27 × 10^4^ tons) agricultural carbon emissions reduction over the study period (1991–2014), whereas the economy factor increased by 50.75% (273.19 × 10^4^ tons) of the total agricultural carbon emissions increment over the study period (1991–2014). The structure factor accounted for 0.97% (5.22 × 10^4^ tons) of the total agricultural carbon emissions increment over the study period (1991–2014). The differences between this stage and the first stage (1991–1995) are that the agricultural carbon emissions decreased by 95.64 × 10^4^ tons in this stage which accounted for −17.77% of the total agricultural carbon emissions increment over the study period (1991–2014). This is the only stage in which agricultural carbon emissions decreased over the study period (1991–2014), and the labour factor was positive (50.22 × 10^4^ tons) for this stage (Table 4).

### Low efficiency and high output (2011–2014)

At this stage, the efficiency factor had a decreasing effect on curbing agricultural carbon emissions, even though it promoted agricultural carbon emissions in 2011 and 2014 which indicated that the effect of the efficiency factor was relatively lower during this stage. As a consequence of the more rapid agricultural economic growth in this stage, the economy factor had an increasing effect on promoting agricultural carbon emissions. Accordingly, the main features of this stage are low efficiency and high output. The reason is that China began to implement a counterpart aid policy in Xinjiang and the agricultural economy developed rapidly, but the development of agricultural science and technology was slow.

The labour factor, the economy factor and the efficiency factor increased the agricultural carbon emissions to various degrees in this stage (Table 5). The labour factor became the most contributing factor to the increase of agricultural carbon emissions, increasing carbon emissions by 150.19 × 10^4^ tons, and the contribution of this effect to the total agricultural carbon emissions increment over the study period (1991–2014) was 27.90%. The economy factor increased carbon emissions by 92.86 × 10^4^ tons, and this accounted for 17.25% of the total agricultural carbon emissions increment over the study period (1991–2014) (Table 5). The efficiency factor increased carbon emissions by 26.76 × 10^4^ tons, and this accounted for 4.97% of the total agricultural carbon emissions increment over the study period (1991–2014) (Table 5).

The structure factor inhibited the agricultural carbon emissions (Table 5), which cut 1.29% (6.96 × 10^4^ tons) of the total agricultural carbon emissions increment over the study period (1991–2014).

Agricultural carbon emissions increased by 262.84 × 10^4^ tons in this stage, accounting for 48.83% of the total carbon emissions increment over the study period (1991–2014), and this stage had the largest increase in agricultural carbon emissions over the study period (1991–2014).

## Discussion

**Figure 6:**
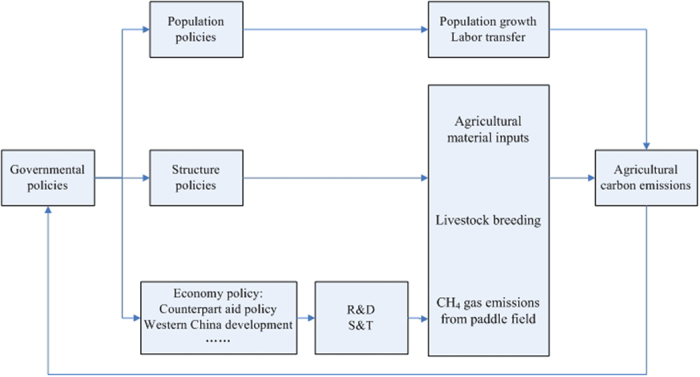
Diagram of the interaction between agricultural carbon emission and governmental policies. R&D indicates research and development, S&T indicates science and technology. Agricultural material inputs include chemical fertilizer, pesticides, plastic sheeting, agricultural diesel, etc.

1. Xinjiang showed different agricultural carbon emission characteristics at different stages. The increase in agricultural carbon emissions was larger in the stages of low efficiency and high output (Tables 3 and 5). The degree of impact on agricultural carbon emissions at the stages of high efficiency and high output and low efficiency and low output depended on the combined effect of planting-animal husbandry carbon intensity and agricultural labour productivity (Tables 1, 2 and 4).
2. The economy factor is the critical factor to promote the increase of agricultural carbon emission, while the main inhibiting factor for agricultural carbon emissions was the efficiency factor in Xinjiang (Fig. 6). Rapid and stable growth of an agricultural economy can help decrease planting-animal husbandry carbon intensity with the progress of agriculture-related science and technology (Table 4). Nevertheless, total agricultural carbon emissions will dramatically increase with a growth in agricultural economy but with a decrease in efficiency (Tables 3 and 5). Our results indicate that it is not sufficient to only improve efficiency when taking agricultural carbon emission reduction as a goal, but the mode and quality of agricultural economic development should also be considered. A moderate agricultural economic growth rate with a higher quality of agricultural economic development and higher efficiency may be a better choice for Xinjiang. Furthermore, technological progress is the most effective way to increase efficiency^2^,^10^, so Xinjiang should accelerate the development of agriculture-related S&T, including agricultural S&T innovation, agricultural S&T guidance and agricultural S&T promotion^14^,^27^,^28^.
3. The structure factor had little effect on agricultural carbon emissions (Tables 1, 2, 3, 4, 5). However, the labour factor became more and more obvious in increasing agricultural carbon emissions (Tables 1, 2, 3, 4, 5) and became the most contributing factor to the increase of agricultural carbon emissions in the last stage (Table 5). The labour factor will be the main driving force of agricultural carbon emissions increase in the future and will also become the difficult point of agricultural carbon emission reduction, especially in ethnic areas in Xinjiang^2^. The reason is that ethnic minority areas in Xinjiang have special population policies that lead to the high natural population growth rate and a large number of labourers being in rural areas^2^. Therefore, Xinjiang should accelerate the transfer of rural labour, control the population growth rate and strengthen the existing family planning policy in ethnic areas.
4. Through the analysis of agricultural carbon emission in Xinjiang since 1991, we found that the effect of government policy on agricultural carbon emissions is very interesting. However, the policies did not act directly on agricultural carbon emission but rather influenced agricultural economic development, the agricultural production structure, and population growth, which indirectly affected agricultural carbon emissions (Fig. 6).
5. In the course of agricultural emission reduction, the three major mechanisms of incentive, tax and compensation should be combined together to influence the agricultural production decision^14^,^29^,^30^,^31^. Xinjiang is lacking in incentive, tax and compensation mechanisms to promote the development of low carbon agriculture. Therefore, Xinjiang should accelerate the establishment and improvement of the three mechanisms.

## Conclusions

In this paper, we analysed the changes in agricultural carbon emissions and identified the main factors that influence agricultural carbon emissions based on different stages in Xinjiang. Agricultural carbon emissions were decomposed into the efficiency factor, the structure factor, the economy factor, and the labour factor by applying the LMDI decomposition method, and we divided the study period into five stages based on the changes in the efficiency factor and the economy factor.

Through the analysis of changes in agricultural carbon emission, Xinjiang’s agricultural carbon emissions showed three stages of change, i.e., continued to rise, declined and continued to rise, during the period of 1991–2014. Xinjiang’s agricultural carbon emissions mainly come from agricultural land use and livestock farming. Xinjiang’s agriculture belongs to the low emission and high efficiency agriculture, with a lower agricultural carbon emission intensity.

Regarding the main factors that influence agricultural carbon emissions based on different stages, the increase in agricultural carbon emissions was larger in the stages of low efficiency and high output. The degree of impact on agricultural carbon emissions at the stages of high efficiency and high output and low efficiency and low output depended on the combined effect of planting-animal husbandry carbon intensity and agricultural labour productivity. The economy factor is the critical factor to promote the increase of agricultural carbon emission, while the main inhibiting factor for agricultural carbon emissions was the efficiency factor in Xinjiang. The labour factor became more and more obvious in increasing agricultural carbon emissions and became the most contributing factor to the increase of agricultural carbon emissions in the last stage (2011–2014). Governmental policies and technological progress were also important factors that affected agricultural carbon emissions.

The development of agricultural S&T and the three major mechanisms (incentive, tax and compensation) are the key to agricultural carbon emission reduction in Xinjiang. On the one hand, Xinjiang should strengthen S&T innovation and promotion, improve the S&T guidance and support of low carbon agriculture, and increase the research and development (R&D) investment^2^,^14^. On the other hand, Xinjiang should establish the three major mechanisms, which should be combined together to influence the agricultural production decision^32^. Through the development of agricultural S&T and the establishment of three major mechanisms, Xinjiang will explore the mode of Circular Eco Low-carbon agriculture.

Of course, due to limitation of available data, further research needs to be performed. Although this study strived to build a perfect index system of agricultural carbon emissions, it still requires further refinement and lacks an analysis for regional differences in Xinjiang. These research topics call for the author to make further studies in the future.

## Additional Information

**How to cite this article**: Xiong, C. *et al*. Changes in agricultural carbon emissions and factors that influence agricultural carbon emissions based on different stages in Xinjiang, China. *Sci. Rep*. **6**, 36912; doi: 10.1038/srep36912 (2016).

**Publisher’s note:** Springer Nature remains neutral with regard to jurisdictional claims in published maps and institutional affiliations.

## Figures and Tables

**Table 1:** Decomposition of carbon emissions during 1992–1995.

Year	Efficiency factor	Structure factor	Economy factor	Labour factor	Total effect
1992	2.91	0.10	9.32	−4.41	7.92
1993	−70.36	0.45	68.81	2.26	1.16
1994	−181.59	3.40	214.82	−5.60	31.03
1995	−120.88	1.21	131.51	6.57	18.41
Total effect	−369.91	5.15	424.46	−1.17	58.53

This table shows the change of efficiency factor, structure factor, economy factor, labour factor and the total effect; its unit is 10^4^ tons.

**Table 2:** Decomposition of carbon emissions during 1996–2002.

Year	Efficiency factor	Structure factor	Economy factor	Labour factor	Total effect
1996	10.90	−0.42	18.59	4.67	33.74
1997	17.80	−0.40	−32.19	49.79	35.00
1998	60.85	−1.88	27.76	−55.18	31.55
1999	95.29	−1.46	−79.04	−3.97	10.83
2000	18.67	0.19	5.29	4.10	28.25
2001	39.86	−2.61	−12.07	0.50	25.69
2002	12.34	−0.27	0.94	6.71	19.71
Total effect	255.70	−6.84	−70.72	6.62	184.77

This table describes factors during the period of 1996–2002.

**Table 3:** Decomposition of carbon emissions during 2003–2005.

Year	Efficiency factor	Structure factor	Economy factor	Labour factor	Total effect
2003	−94.21	−22.47	156.13	10.23	49.68
2004	11.13	−1.53	19.45	11.96	41.01
2005	−4.36	6.23	25.67	9.54	37.08
Total effect	−87.45	−17.77	201.25	31.73	127.77

This table describes factors during the period of 1996–2002.

**Table 4:** Decomposition of carbon emissions during 2006–2010.

Year	Efficiency factor	Structure factor	Economy factor	Labour factor	Total effect
2006	24.00	−1.52	−14.63	12.71	20.56
2007	−124.17	0.92	55.91	4.23	−63.10
2008	−58.13	−10.37	−3.28	4.09	−67.70
2009	−33.47	1.51	26.28	10.76	5.07
2010	−232.50	14.69	208.91	18.44	9.53
Total effect	−424.27	5.22	273.19	50.22	−95.64

This table describes factors during the period of 2006–2010.

**Table 5:** Decomposition of carbon emissions during 2011–2014.

Year	Efficiency factor	Structure factor	Economy factor	Labour factor	Total effect
2011	24.20	−3.39	−34.16	44.31	30.97
2012	−24.85	0.46	54.27	52.88	82.76
2013	−5.46	−3.38	33.76	27.81	52.74
2014	32.86	−0.65	38.97	25.19	96.37
Total effect	26.76	−6.96	92.86	150.19	262.84

This table describes factors during the period of 2011–2014.
